# Zeldes, N. ‘Navigating the cultures of health care and health insurance: Highly skilled migrants in the US’. London: UCL Press. 2023. pp. 202. 216. £20 (pbk), £40.00 (hbk). ISBN: 978-1-80008-365-3

**DOI:** 10.1111/1467-9566.13773

**Published:** 2024-06

**Authors:** Matthew S. Hanchard

**Affiliations:** Research Fellow, iHuman, University of Sheffield

Amidst a turn towards neoliberalism, transnational mobility has become an expectation of and for many highly skilled workers, raising uncertainty in many aspects of their everyday lives. It is here that Zeldes focusses on one aspect of this turn - the trials, tribulations, and overall experience of navigating healthcare and health insurance cultures for highly skilled migrants moving into the USA. To qualify and ground the book’s theoretical claims, Zeldes meticulously details her empirical approach. The book is based on 65 interviews, analysed using grounded theory. She also undertook concordance (collocation) and frequency analyses of a sizeable narrative corpus, with her analysis steeped in an ethnographic immersion in the greater Washington, DC metropolitan area (inclusive of suburbs in Maryland and Virginia) conducted between 2015-2017. Here, further work might usefully extend Zeldes’ by conducting controversy analysis or network analysis to extract political underpinnings from narratives and discourse in the corpus in more depth than token count and/or frequency distributions.

Rather than looking at health inequity and socio-economic exclusion – already well-trodden ground – Zeldes instead looks at highly skilled migrants (and their spouses) entering the USA from three countries (Germany, India, and Japan). Thus, the book undoubtedly adds novelty and value to the field, offering insights into a largely overlooked healthcare topic. Similarly, while Zeldes extends research undertaken for her PhD, in doing so the book does not follow the rote chapter format of a thesis, breaking down the analyses into thematic chapters instead – which makes for a far more interesting read.

Throughout, Zeldes demonstrates that highly skilled migrants moving to the USA draw on past experiences to navigate the complex and conglomerated US health care system(s). Doing so shades their expectations and interactions with physicians and dentists, with nuanced cultural differences in highly skilled migrant patients’ expectations and forms of healthcare consumption. In this, Zeldes contributes to discussion about US immigrants receiving disproportionately less healthcare (Ku, 2009) whilst harbouring longer life-expectancies and better health outcomes than *jus soli* citizens (Viruell-Fuentes, 2007). Here, Zeldes’ work highlights a need to break down claims with greater granularly by looking at differing groups of immigrants’ past healthcare experiences.

As a secondary argument, Zeldes explains how highly skilled migrants adapt to US healthcare systems by disentangling concerns about healthcare delivery and coverage (i.e., by insurance, co/payment, and/or reimbursement) in different ways. Her argument follows that cultural nuance on expectations give rise to such differences. Highly skilled migrants from Germany and Japan, for instance, see value in health insurance - especially German ones – but remain unable to fully ‘…understand the political debates on health insurance coverage [in the US]…’ seeing instead more ‘…advantages of the insurance plans of their respective home countries’ (p. 72). Those from India, by contrast, ‘…often find health insurance unnecessary’ (p. 73); a finding that may pique the interests of health economists and insurance brokers alike in developing and selling future health insurance products.

Elsewhere, Zeldes provides useful background context. In chapter three, for instance, she offers an overview of the US health care insurance landscape, comparing it with those in Germany, India, and Japan. Perhaps as a product of its time, this may have benefitted from more recent discussion of the US Inflation Reduction Act 2022 and the emergence of pharmacy benefit fund managers. Similarly, the research underpinning the book took place just before the impact of a global coronavirus pandemic took hold on health insurance prices. The latter is mentioned within a footnote in chapter 4, but with perhaps a little too much brevity. Here, a short section outlining changes to the USA health insurance since the fieldwork period might have added depth. Yet serendipitously, its omission does provide scope for further comparative study to extend the book. Thus, the book (chapter 2 in particular) generates a much-needed primer that will no doubt become a staple reading on many medical sociology and medical anthropology courses.

In sum, this book provides a novel set of findings and a main argument that will undoubtedly be of interest for public health and healthcare policymakers, and medical anthropologists and medical sociologists. Zeldes’ arguments are well-qualified and grounded within a rigorous methodology, with aliquot parts (i.e. background context sections in chapter 2) adding wider value. In tone, however, the book feels more like the start of a conversation than a definitive conclusion and opens several fruitful avenues for further research. it meets its aim well, though, generating a better understanding of the complex ways in which differing cultural aspects and past experiences affect highly skilled migrants’ expectations and uptake of healthcare and healthcare insurance.
